# The Assessment of Vitamin D Levels in Pregnant Women is not Associated to Fetal Growth Restriction: A Cross Sectional Study

**DOI:** 10.1055/s-0041-1735158

**Published:** 2021-11-16

**Authors:** Vivian Macedo Gomes Marçal, Francisco Lázaro Pereira Sousa, Silvia Daher, Raquel Margiotte Grohmann, Alberto Borges Peixoto, Edward Araujo Júnior, Luciano Marcondes Machado Nardozza

**Affiliations:** 1 Department of Obstetrics, Escola Paulista de Medicina, Universidade Federal de São Paulo, São Paulo, SP, Brazil; 2 Department of Obstetrics and Gynecology, Faculdade de Ciências Médicas de Santos, Santos, SP, Brazil; 3 Department of Gynecology and Obstetrics, Universidade Federal do Triângulo Mineiro, Uberaba, MG, Brazil; 4 Gynecology and Obstetrics Service, Hospital Universitário Mário Palmério, Universidade de Uberaba, Uberaba, MG, Brazil

**Keywords:** pregnancy, maternal serum levels, vitamin D, small for gestational age, fetal growth restriction, gestação, concentração sérica materna, vitamina D, pequeno para idade gestacional, restrição de crescimento fetal

## Abstract

**Objective** To assess maternal serum levels of vitamin D in fetuses appropriate for gestational age (AGA), small for gestational age (SGA), and with fetal growth restriction (FGR) according to estimated fetal weight (EFW).

**Methods** This cross-sectional study included 87 pregnant women between 26 and 36 weeks of gestation: 38 in the AGA group, 24 in the SGA group, and 25 in the FGR group. Maternal serum vitamin D levels were assessed using the chemiluminescence method. The Fisher exact test was used to compare the results between the groups.

**Results** The mean ± standard deviation (SD) of maternal age (years) and body mass index (kg/m ^2^ ) in the AGA, SGA, and FGR groups were 25.26 ± 8.40 / 26.57 ± 4.37; 25.04 ± 8.44 / 26.09 ± 3.94; and 25.48 ± 7.52 / 26.24 ± 4.66, respectively ( *p* > 0.05). The maternal serum vitamin D levels (mean ± SD) of the AGA, SGA, and FGR groups were 22.47 ± 8.35 ng/mL, 24.80 ± 10.76 ng/mL, and 23.61 ± 9.98 ng/mL, respectively, but without significant differences between the groups ( *p* = 0.672).

**Conclusion** Maternal serum vitamin D levels did not present significant differences among pregnant women with AGA, SGA, or FGR fetuses between 26 and 36 weeks of gestation according to EFW.

## Resumo

**Objetivo** Avaliar o nível sérico materno de vitamina D em fetos adequados para idade gestacional (AIG), pequenos para idade gestacional (PIG) e com restrição de crescimento (RCF) de acordo com a estimativa de peso fetal (EPF).

**Métodos** Realizou-se um estudo transversal envolvendo 87 gestantes entre 26 e 36 semanas, sendo: 38 do grupo AIG, 24 do grupo PIG e 25 do grupo RCF. A dosagem sérica materna de vitamina D foi realizada pelo método de quimiluminescência. Para as comparações entre os grupos, utilizou-se o teste exato de Fisher.

**Resultados** A média ± desvio-padrão (DP) da idade materna (anos) e do índice de massa corporal (kg/m ^2^ ) nos grupos AIG, PIG e RCF foram 25,26 ± 8,40 / 26,57 ± 4,37; 25,04 ± 8,44 / 26,09 ± 3,94; e 25,48 ± 7,52 / 26,24 ± 4,66, respectivamente ( *p* > 0,05). A concentração sérica materna de vitamina D (médias ± desvios-padrão) dos grupos AIG, PG e RCF foram 22,47 ± 8,35 ng/ml; 24,80 ± 10,76 ng/ml; e 23,61 ± 9,98 ng/ml, respectivamente, contudo, sem diferenças significativas entre os grupos ( *p* = 0,672).

**Conclusão** A concentração sérica materna de vitamina D não apresentou diferenças significantes entre gestantes com fetos AIG, PIG ou RCF entre 26 e 36 semanas de acordo com a EPF.

## Introduction

Fetal growth restriction (FGR) affects ∼ 5 to 10% of pregnancies and is the second obstetric complication with higher perinatal mortality, responsible for ∼ 30% of stillbirths, as well as a cause of higher frequency of premature births and intrapartum asphyxia. 1 Small for gestational age (SGA) fetuses are those with prediction of weight below the 10 ^th^ percentile for gestational age, without impairing their genetic potential for growth. 2

Currently, the classification of FGR follows the Delphi consensus, in which the fetuses are classified with early (< 32 weeks) and late fetal growth restriction (≥ 32 weeks), excluding congenital anomalies. 3 Hypertrophy of fetal cells begins approximately at 32 weeks, and the importance given to the abdominal circumference (AC) is justified by the reduction of the liver, with reduction of glycogen storage associated with a decrease in abdominal fatty tissue. 4

Vitamin D is a steroid involved in intestinal absorption and regulation of calcium homeostasis and is essential for the formation and maintenance of healthy and strong bones. Vitamin D deficiency may be due to inadequate exposure to the sun, inefficient food intake, decrease in absorption, and abnormal metabolism. 5 Recent studies have related vitamin D deficiency during pregnancy to preeclampsia, 6 gestational diabetes mellitus, 7 and prematurity; 8 yet, the relationship with FGR or SGA fetuses remains uncertain.

Bodnar et al. 9 sought to elucidate the association between maternal serum concentrations of 25-hydroxyvitamin D (25(OH)D) and the risk of SGA fetuses. They observed a relationship between maternal 25(OH)D serum level and risk of SGA in white women but not in black women, suggesting that vitamin D has a complex relationship with fetal growth that may vary according to race. Gernand et al. 10 evaluated the association between maternal 25(OH)D levels and increased risk of placental insufficiency and observed a relationship between 25(OH)D and vascular damage, with 25(OH)D ≥ 80 nmol/L associated with 49% lower risk of FGR in male newborns.

Therefore, the objective of the present study is to assess the vitamin D serum levels of mothers with SGA and FGR fetuses, comparing them with those of mothers with fetuses appropriate for gestational age (AGA) between 26 and 36 weeks of gestation according to estimated fetal weight (EFW).

## Methods

A cross-sectional study was conducted between November 2016 and July 2019. The study was approved by the local research ethics committee under protocol No. 2.004.104, and all participants signed an informed consent form. The study was conducted at two university hospitals.

The inclusion criterion was pregnancy with a single fetus between 26 and 36 weeks of gestation, and the exclusion criteria were women in labor, fetuses with congenital anomalies detected on ultrasound, and chronic diseases such as hypertension, diabetes mellitus, autoimmune diseases, and heart diseases. Gestational age was determined by the date of the last menstrual period (LMP) and confirmed by ultrasonography performed up to 13 weeks.

The pregnant women were divided into 3 groups: 1) AGA (control); 2) SGA; and 3) FGR. Appropriate for gestational age was defined if the EFW was between 10 ^th^ and 90 ^th^ percentile according to the respective gestational age, 11 following normal values of pulsatility index (PI) of the umbilical artery (UA), PI of the middle cerebral artery (MCA) and mean PI of the uterine artery (UtA). Fetuses were considered to have early-onset FGR when the gestational age was < 32 weeks and the following criteria were present: EFW or AC < 3 ^rd^ percentile for the gestational age or absent end-diastolic flow in the UA; EFW or AC < 10 ^th^ percentile for the gestational age, associated with a mean PI of the UtA or PI of the UA > 95 ^th^ percentile for the gestational age. 3 Fetuses were considered to have late-onset FGR when the gestational age was > 32 weeks and the following criteria were present: EFW or AC < 3 ^rd^ percentile for the gestational age; EFW or AC < 10 ^th^ percentile for the gestational age, associated with a mean PI of the UA > 95 ^th^ percentile for the gestational age, cerebro-placental ratio (CPR) < 5 ^th^ percentile for the gestational age, or AC/EFW ratio crossing centiles > 2 quartiles on growth centiles. 3 Fetuses were considered SGA when EFW was between 3 ^rd^ and 10 ^th^ percentile and the criteria for early- and late-onset FGR diagnosis were not met.

The ultrasound examinations were performed using a diagnostic WS80 Ultrasound System (Samsung Corp., Seoul, South Korea) by experienced examiners. Biometric measurements and EFW were determined, according to the equation by Hadlock et al. 12 The Doppler parameters of the MCA and UA arteries were evaluated according to the curve of Arduini and Rizzo. 13 These were considered altered when the MCA PI < 5 ^th^ percentile and/or the UA PI > 95 ^th^ percentile for gestational age. The UtA Doppler parameters were evaluated according to the curve reported by Gómez et al. 14 and were considered abnormal when the mean PI > 95 ^th^ percentile for gestational age. The volume of the amniotic fluid was evaluated by the four quadrants technique, according to the amniotic fluid index (AFI), 15 with AFI < 5 cm being considered oligohydramnios.

Maternal blood samples were collected during prenatal consultations only once and when the EFW < 10 ^th^ percentile by ultrasound evaluation. Peripheral venous punctures were performed by two trained investigators, and the material was homogenized by inversion 5 to 8 times, accommodated in a sealed tube, and kept in a vertical position for 30 minutes. After complete blood coagulation, centrifugation was performed at 3,000 rpm for 15 minutes, and the samples were sent for laboratory analysis.

The ADVIA Centaur Vitamin D Total test (Siemens Healthineers, Erlangen, Germany) was used in the in vitro quantitative determination of total vitamin D 25(OH) in human serum and plasma. This is an 18-minute single pass competitive immunoassay using mouse monoclonal acridine ester (AE) labeled anti-vitamin D 25(OH) antibody and a fluorescein-labeled vitamin D analog. The ADVIA Centaur and ADVIA Centaur XP systems automatically perform the following steps: 1) dispenses 20 µL of sample into a cuvette and incubates for 15 seconds; 2) dispenses 200 µL auxiliary reagent and incubates for 4.5 minutes at 37° C; 3) dispenses 50 µL of lite reagent and incubates for 5.5 minutes at 37° C; 4) dispenses 100 µL of solid phase and 50 µL of auxiliary container reagent and incubates for 2.75 minutes at 37° C; 5) separates the solid phase from the mixture and aspirates unbound reagent; 6) washes the cuvette with wash solution 1; and 7) dispenses 300 µl of acid reagent and base reagent to initiate the chemiluminescent reaction.

The ADVIA Centaur systems communicate the results by e-mail, which, according to an analysis of the literature, recommends the following classification for 25(OH)D levels: 1) deficiency < 20 ng/mL (50 nmol/L); 2) insufficiency between 20 and 30 ng/mL (50–75 nmol/L); 3) sufficiency between 30–100 ng/mL (75–250 nmol/L); and 4) toxicity > 100 ng/mL (250 nmol/L).

A power analysis was performed to calculate the sample size on the basis of the Cohen effect of 0.35 to achieve a power of 80% and an α of 5% to detect the differences in the evaluated parameters. 16 Using the software G 3.1, the results suggested at least a sample size of 84 fetuses distributed homogeneously.

The data were transferred to an Excel 2010 spreadsheet (Microsoft Corp., Redmond, WA, USA) and analyzed with the SPSS for Windows, Version 15.0 (SPSS Inc., Chicago, IL, USA). The variables analyzed in the study were acquired on the day of the prenatal care consultation, when a questionnaire was applied with the following data: maternal age (years), weight (Kg), height (m), body mass index (BMI) (m/Kg ^2^ ), gestational age (weeks), consumption of fish (150 g at least 3 times a week), sun exposure (at least 20 minute per day), sun protection (yeas or no), vitamin D replacement therapy (yes or no) and smoking (at least 1 cigarette per day). From the point of view of inferential statistics, to compare the groups (AGA, SGA, and FGR) with regard to the numerical variables of the study, we applied the analysis of variance model with a fixed factor and Tukey multiple comparisons method. For categorical variables, the Fisher exact test was used. In all analyses, a significance level of *p* < 0.05 was set.

## Results

Initially, blood samples from 100 pregnant women were collected; however, 13 samples were excluded due to the unavailability of the material at the time of analysis. Therefore, the final samples included 38 from the AGA group, 24 from the SGA group, and 25 in the FGR group. Table 1 presents the descriptive analysis of maternal characteristics of the three groups.

**Table 1:** Comparison of numerical maternal variables in all three groups evaluated

	AGA ( *n* = 38)				SGA ( *n* = 24)				FGR ( *n* = 25)				*p* -value *
	Mean	SD	Min	Max	Mean	SD	Min	Max	Mean	SD	Min	Max	
Age (years)	25.3	8.4	15.0	42.0	25.0	8.4	15.0	41.0	25.5	7.5	15.0	42.0	0.983
GA (weeks)	31.1	2.9	26.0	35.9	33.0	2.6	27.7	36.7	32.2	3.2	26.3	36.0	0.556
Maternal weight (Kg)	69.4	14.6	49.3	105.8	65.3	11.0	51.0	84.6	65.2	13.1	39.0	85.2	0.659
Maternal height (m)	1.6	0.1	1.5	1.8	1.6	0.1	1.5	1.8	1.6	0.1	1.5	1.7	0.690
Maternal BMI (Kg/m ^2^ )	26.6	4.4	19.8	37.1	26.1	3.9	20.7	35.2	26.2	4.7	16.4	34.6	0.922
EFW (grams)	1725	526,6	958	2788	1678,0	408,7	879	2261,0	1409	455,4	630	2053	0.030

Abbreviations: AGA, appropriate for gestational age; BMI, body mass index; EFW, estimated fetal weight; FGR, fetal growth restriction; GA, gestational age at blood sample collection; SD, standard deviation; SGA, small for gestational age.

* Tukey's multiple comparisons, *p* < 0.05.

Regarding categorical variables, the consumption of fish was low in all three groups. In relation to sunlight exposure, a more balanced result was observed, with 59.8% of pregnant women not sunbathing regularly. In relation to vitamin D and smoking, 94.3% and 86.2%, respectively, did not supplement this vitamin and did not smoke. No pregnant woman reported using sunscreen. Table 2 shows the comparison between the groups, with no statistical differences between them in any of the categorical variables.

**Table 2:** Comparison of maternal variables and vitamin D level in the three analyzed groups

	AGA ( *n* = 38)	SGA ( *n* = 24)	FGR ( *n* = 25)	*p* -value
Fish consumption	7 (18.4%)	1 (4.2%)	5 (20%)	0.233 *
Exposure to sunlight	15 (39.5%)	9 (37.5%)	11 (44%)	0.925 *
Smoking	8 (21.1%)	2 (8.3%)	2 (8%)	0.325 *
Vitamin D supplement	3 (7.9%)	1 (4.2%)	1 (4%)	0.999 *
Vit D level (ng/ml)	22.5 ( ± 8.3)	23.6 ( ± 9.9)	24.8 ( ± 10.8)	0.672 **

Abbreviations: AGA, appropriate for gestational age; FGR, fetal growth restriction; SGA, small for gestational age, Vit, vitamin.

* Fisher exact test: frequency (centile).

** ANOVA: mean (standard deviation), *p* < 0.05.

The mean ( ± SD) levels of maternal serum vitamin D for the AGA, SGA, and FGR groups (22.47 ± 8.35 ng/mL, 24.80 ± 10.76 ng/mL, and 23.61 ± 9.98 ng/mL, respectively) showed no significant differences between the groups ( *p* = 0.672) ( Table 2 ). The results were compatible with vitamin D insufficiency (20–30 ng/mL) in the 3 groups.

**Fig. 1:**
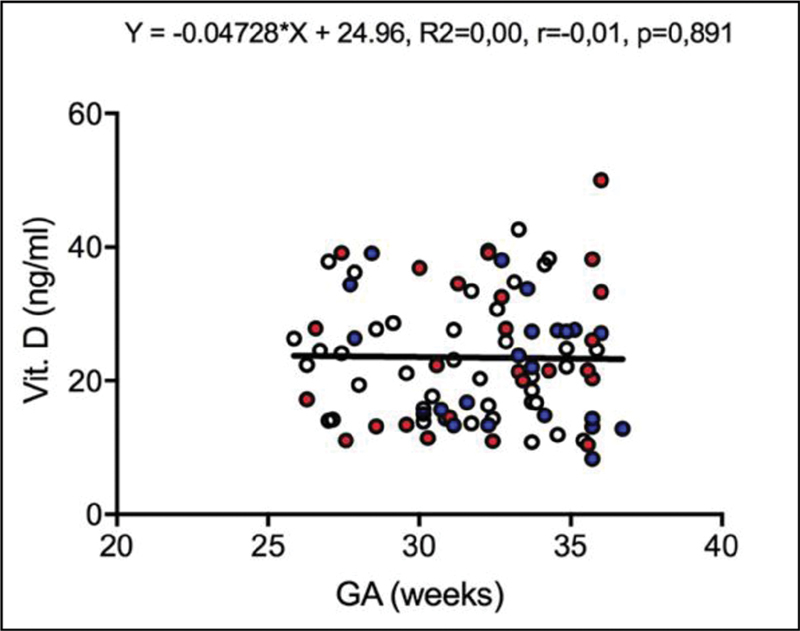
Scatter plot of the vitamin D level (ng/ml) according to gestational age (weeks) in all pregnant women included in the study. Open dots: adequate for the gestational age; blue dots: small for the gestational age; red dots: fetal growth restriction. Pearson correlation coefficient, *p* < 0.05.

**Fig. 2:**
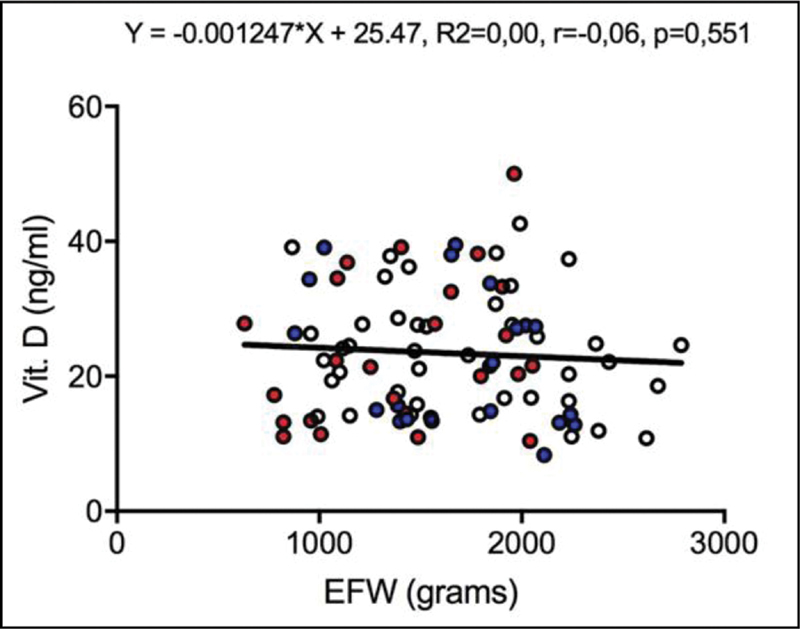
Scatter plot of the vitamin D level (ng/ml) according to estimated fetal weight (grams) in all pregnant women included in the study. Open dots: adequate for the gestational age; blue dots: small for the gestational age; red dots: fetal growth restriction. Pearson correlation coefficient, *p* < 0.05.

Considering all cases included in the study, there was no significant correlation between vitamin D levels and gestational age (r = - 0.01, *p* = 0.891) ( Fig. 1 ). Furtheremore, there was no significant correlation between vitamin D levels and EFW (r= -0.06, *p* = 0.551) ( Fig. 2 ).

## Discussion

The inability of a fetus to attain the weight corresponding to its genetic potential increases morbidity and perinatal mortality; thus, FGR and obstetrical pathology must be diagnosed and managed early and adequately. 17 Fetal growth restriction increases the risk of long-term sequelae, such as coronary heart disease, diabetes mellitus type 2, arterial hypertension, and metabolic syndrome. 18 19 20 Therefore, the knowledge of extrinsic predisposing factors could help in the early diagnosis of this pathology. Accordingly, we evaluated the effect of maternal parameters such as BMI on FGR, as a study found that pregnant women with FGR fetuses have low BMI compared with pregnant women with AGA fetuses. 21

Classic obstetric complications have been associated with serum Vitamin D levels, 22 23 24 25 even though the challenge of establishing a value specifically attributable to the pregnancy and puerperal period is recognized. However, the tendency of the available literature is to adopt indices from the non-pregnant population, which as can be seen in a recent systematic review with meta-analysis of 54 articles. 26 Furthermore, we did not find any significant correlation between vitamin D level and gestational age, as well vitamin D level and EFW. This condition supported our choice for the Vitamin D scores that classified the group of women studied.

Vitamin D insufficiency is associated with obstetric pathologies such as preeclampsia and diabetes mellitus. 6 7 During pregnancy, supplementation of this vitamin can be a viable strategy to prevent fetuses with low birth weight and SGA; 22 for this reason, the present study aimed at assessing the correlation of vitamin D with fetal growth.

Vitamin D deficiency in pregnant women is a major concern due to the risk of adverse obstetric pathologies and perinatal outcomes. 23 24 The level of 25(OH)D, which is the main form of vitamin D storage in humans, can, therefore, be measured in maternal blood to determine overall vitamin D status.

In the current study, low levels of vitamin D were observed in pregnant women between 26 and 36 weeks of gestation, living in a tropical country like Brazil, with abundant sunshine. In the south of China, which also has a tropical climate and where women were believed to have sufficient exposure to ultraviolet B radiation and regular vitamin supplementation in prenatal care, a high prevalence of low levels of vitamin D between 16 and 20 weeks of gestation was also observed. 25 No significant differences in adverse perinatal outcomes were observed between pregnant women with different vitamin D levels, except for a higher prevalence of gestational diabetes mellitus and preterm delivery in women with high serum vitamin D levels. 25

The present study demonstrated a high prevalence (75.9%) of low serum vitamin D levels (deficient and insufficient levels) in pregnant women evaluated, regardless of the group assessed. A systematic review and meta-analysis, which included 54 eligible studies, reported that vitamin D deficiency (< 30 ng/mL) was associated with SGA, 26 unlike our study, which did not identify this association. This systematic review also identified the occurrence of preterm birth and deficits in mental development and language when vitamin D insufficiency was present. 26

In this study, five women received vitamin D supplementation, with three pregnant women having AGA fetuses, one having a SGA fetus, and the other one having a FGR fetus. In this study, we did not assess the differences associated with the skin color of pregnant women.

As limitations of the study are cross-sectional character, which did not evaluate the neonatal outcomes, thus making it impossible to compare with birthweight. Furthermore, although the sample size is within the statistical calculation, the small number of cases may have impacted our results. Futures studies with a higher number of cases are necessary to prove our results.

## Conclusion

In summary, maternal serum concentration of vitamin D assessed between 26 and 36 weeks of pregnancy showed no significant differences between cases identified through EFW as AGA, SGA, or FGR.
